# Smoking Patterns and Smoking Cessation Willingness—A Study among Beneficiaries of Government Welfare Assistance in Poland

**DOI:** 10.3390/ijerph14020131

**Published:** 2017-01-27

**Authors:** Katarzyna Milcarz, Teresa Makowiec-Dąbrowska, Leokadia Bak-Romaniszyn, Dorota Kaleta

**Affiliations:** 1 Department of Tobacco Control, Preventive Medicine Department, Medical University of Lodz, Lodz 90-752, Poland; dkaleta@op.pl; 2 Department of Work Physiology and Ergonomics, Nofer Institute of Occupational Medicine, Lodz 91-348, Poland; tmd@imp.lodz.pl; 3 Department of Nutrition in Digestive Tract Diseases, Medical University of Lodz, Lodz 93-338, Poland; leokadia.bak-romaniszyn@umed.lodz.pl

**Keywords:** tobacco control, cigarettes, smoking cessation, electronic cigarettes, disadvantaged groups, low socioeconomic status population

## Abstract

This study examines the prevalence and tobacco use patterns among adult social assistance beneficiaries and their interest in quitting. The results are based on data collected in a cross-sectional survey conducted among adults in the Piotrkowski district. A sample of 3636 social assistance beneficiaries produced a total of 1817 respondents who completed face-to-face questionnaires. Overall, 37.1% of the respondents, including 52.8% men and 29.6% women, were current smokers. Over one third of the smokers reported their willingness to quit. In the study population, several characteristics were significantly associated with the current daily smoking: male gender, low educational attainment, unemployment or temporary employment, lack of awareness of smoking-associated health risks, use of e-cigarettes, and exposure to environmental tobacco smoke (ETS). The intention to quit smoking among the daily smokers was positively correlated with their awareness of smoking-associated health risks, lack of previous quit attempts, and low exposure to ETS. Smoking prevalence among social assistance recipients tends to be higher than in the general population, but more than half of the smokers are willing to quit. There is an urgency to develop policies tailored to the needs of these disadvantaged population groups.

## 1. Introduction

Tobacco use increases the risk of death from many diseases: cancer, ischemic heart disease, chronic obstructive pulmonary disease (COPD), and stroke are the most common ones [1]. Because smoking is so harmful, differences in smoking prevalence across the population translate into major differences in mortality and morbidity rates. Smoking is the single most important driver of health inequalities across Europe [1]. Mortality rate inequalities caused by tobacco-related diseases in recent years have provided for 22% of the general mortality inequalities for males and 6% for females [2,3]. Tobacco-related deaths provide for over a half of the general mortality inequalities between males with a higher and lower socioeconomic status in Poland [2,4]. Despite the observed reduction of the frequency of smoking in Europe, the process has not been as rapid as could be expected, and the degree of progress varies [5]. In general, the prevalence of smoking across the European countries is greater among people coming from less favorable socioeconomic backgrounds—these tend to start smoking at an earlier age, smoke more cigarettes per each 24-h period, and are less successful at quitting smoking compared to those enjoying a better life situation [6,7,8,9,10,11,12]. Reduction in tobacco consumption is one of the biggest challenges for public health. The prerequisite for a viable action against the damages caused by smoking is the implementation of policies and programs that take the social determinants of health and health inequalities into account [5]. Scientists and researchers currently have access to an extensive knowledge base, comprehensive strategies of proven effectiveness, as well as new technologies which may support initiatives undertaken at local, national and European levels to mitigate health inequalities [13]. One must not forget, however, that tobacco control policies have different impacts on different groups [5]. Health inequalities may be reduced through measures that have greater impact on smokers in higher prevalence groups [5,14]. In practice, this means prioritizing population-level interventions which disadvantaged are more sensitive to and targeting interventions on these smokers. In order to inform stakeholders and improve existing health policy and health care as well as to develop and implement effective tobacco control interventions, detailed data must be collected on prevalence, the determinants of the tobacco epidemic, and the effectiveness of the programs implemented not only in relation to the general population, but also in relation to the disadvantaged populations [5,15,16]. If population diversity is not taken into account, programs and service delivery are likely to be inequitable [17]. A number of epidemiological, health and medical studies showed under-representation of socioeconomically disadvantaged respondents, which was primarily due to barriers in their sampling, recruitment, participation, and retention, and samples based on the general population were rarely sufficient to adequately examine outcomes according to social group [17]. A systematic review by Boneski et al. listed numerous barriers to disadvantaged group participation in medical research, including difficulties in identifying and sampling the hidden populations, mistrust of medical research and the researcher, cultural or language barriers, low literacy, and low education levels [17,18]. Internationally, there is some evidence on smoking among disadvantaged adults, including social and community service organization clients [8,9,10,11,12,18]. Even though a number of monitoring activities are taking place to examine smoking, including the Global Adult Tobacco Survey (GATS), the Prevalence and Control of Cardiovascular Risk factors in Poland Survey (NATPOL), the Multi-center All Polish Health Survey-the WOBASZ Project and the Cardiovascular Diseases Prevention Program (POLSCREEN) studies, no study focusing exclusively on smoking patterns among the socially disadvantaged individuals has been conducted in Poland to date [19,20,21,22,23]. 

Social aid in Poland is administered by the local and national governments [24]. The current form of social aid has been in place since 1 May 2004. The right to benefits under the system is given to individuals and families who are unable to cope with difficult life situations using their own empowerment, resources and abilities. The Social Welfare Act distinguishes between monetary and non-monetary benefits that can be awarded to beneficiaries. The main monetary benefits cover providing an income at the social intervention threshold for individuals with no or low income, to the elderly and the disabled and providing an income at the social intervention threshold to individuals and families with low income who are in need of temporary support. In some cases money-based benefits may be provided, in particular, to cover all or part of the cost of the purchase of food, medicines and treatment, fuel, clothing, essential household items expenses. Non-monetary assistance includes providing professional assistance of social workers to families affected by social pathologies. Non-monetary benefits include also: social work, credit ticket, social insurance contributions, health insurance contributions, benefit in kind e.g., assistance to reach financial self-dependence, funeral allowance, special guidance, crisis intervention, assistance in form of providing shelters, meals, clothes, attendance service provided at homes, assistance in gaining adequate housing conditions including social dwelling, in gaining employment and for running one’s own household. In 2014 assistance provided by the social aid organizations was offered to 1.2 million households and almost 3 millions individuals (family members and the homeless). The proportion of households and individuals who received social aid to the overall number of households and population in Poland was 8.4% and 7.7% respectively. The vast majority of individuals receiving social aid (99%) were household members, with the remaining 1% being homeless people. Social aid is predominantly offered to unemployed working age population, with half of the group being occupationally passive individuals. Young people under 18 represent 35% of the beneficiaries. The elderly population, i.e., people aged 64 and over, represents 10% of the beneficiaries of social aid. Individuals with an education lower than secondary school use social aid over three times more frequently than people with secondary and higher education [24]. Unemployment, inability or unwillingness to undertake a job is the key poverty factor. The primary objectives of social aid include the following: supporting families and individuals with coping with difficult life situations, leading them, as much as feasible, to life independence and empowering them to live their lives in agreement with the general notion of human dignity. Taking into account the large group of social aid beneficiaries and the broad range of competencies they have, social aid institutions and their employees can potentially be the crucial liaison while delivering anti-tobacco programs to those in need.

The main purpose of this work is to examine the prevalence and tobacco use patterns in the adult population of social assistance beneficiaries and their interest in quitting. Predictors of daily smoking and willingness to quit have also been part of the study. 

## 2. Material and Methods

### 2.1. Characteristics of the Piotrkowski District

According to the state data for the year 2013, there were 91,618 residents, including 45,223 men and 46,395 women, living within the Piotrkowski district, with more than 90% of the residents representing rural areas. In 2013, about 9% of the residents required the support of social assistance institutions as a result of lacking resources, and poverty affected 11,867 people in 4336 families [24]. These persons receive aid from the local government welfare assistance institution. For the purposes of this study, the poverty threshold as adopted by the social assistance institutions was used (incomes lower than specific poverty lines as identified in the Social Assistance Act), as reflected in the applicable regulations [24]. This approach to the poverty line is binding upon all district level social assistance institutions. In the group of 11,867 social assistance beneficiaries, 3636 residents were aged 18–59. An analysis completed by the United Nations Development Program (UNDP) identified the Piotrkowski district as 11th among all 314 rural districts with the lowest indicators of social development in Poland [25]. The Local Human Development Index (LHDI), covering three indicators: Health Index, Education Index, and Welfare Index, was 25.97, with the Health Index (HI) = 26.50, whereas the discussed indicators for the Lodzkie voivodeship were 39.28 and 31.48 respectively [26]. Considering the existing health inequalities in Poland and the low level of attention to health inequalities in prevention planning activities of the Polish regions, the Polish government, in cooperation with the Norwegian Directorate of Health Affairs, implemented Project PL-13 “Reducing social inequalities in health” [27]. The project was funded by the Norwegian Financial Mechanism 2009–2014 and the Polish state (www.eeagrants.org). The program consisted of a pre-defined project contributing to the development of cross-sectoral strategies and tools for reducing health inequalities, and pilot projects for comprehensive measures in the field of public health in local communities. 156 Polish powiats (districts) showing the highest overall mortality rates and mortality arising from the top five causes of death were entitled to participate in the project. One of the districts qualified for the project was the Piotrkowski district (Piotrków Trybnalski) [27,28]. The program aimed at reducing inequalities in health among the residents of the Piotkowski district through an initiative known as “Your heart is your life”, which covered a number of actions (including epidemiological studies aimed at understanding the health status, health behaviors of the local community, as well as a broad number of activities in the field of health promotion) targeting socioeconomically disadvantaged people. For the purposes of this study, the definition of socioeconomically disadvantaged (SD) population covers persons with the minimum income not greater than 634 PLN per month for single persons and 514 PLN per month for family members [25]. The data used in the current analysis were collected as a part of the project “Your heart is your life”. 

### 2.2. Design and Sample

A cross-sectional study was conducted between October 2015 and February 2016 among a group of adults aged 18–59, both males and females, who at the time resided in the Piotrkowski district and received aid offered by the local social assistance organizations—a list of the recipients was obtained from the local government. The study covered all those registered in the local government welfare assistance institutions who met the inclusion criteria and agreed in writing to participate in the study. Written informed consents were obtained from all study participants. Before the study was actually carried out, the Bioethics Committee of the Medical University in Lodz issued a positive opinion on the project (Project Identification Code: RNN/243/15/KE).

### 2.3. Participant Survey

The questions included in the survey form cover a number of important issues, such as smoking, the use of e-cigarettes, physical activity, dietary habits, and alcohol consumption among adults. The questionnaire was adapted from the Multi-Centre National Population Health Examination Survey (WOBASZ) [20]. Survey forms were administered by qualified interviewers at respondents’ places of residence during face-to-face interviews. The fieldwork was preceded by several training sessions for all survey staff, and followed by a pre-test. Data collection was coordinated and supervised by a principal investigator, study coordinator and two field supervisors.

The following data were collected: gender (male or female), age (years), marital status, education, employment, subjective assessment of monthly income, subjective assessment of health condition, declared health problems, and alcohol consumption. Marital status was classified as single, married, divorced, or widowed. Educational level was classified as: primary education, vocational education, secondary education, and higher education. The measure of economic activity classified respondents as currently employed with a permanent job, temporarily employed, retired, disability pensioners, students, currently without a permanent or part time job, or unemployed. Subjective health condition was rated as follows: fair, rather fair, neither fair nor poor, rather poor, poor. Monthly income was assessed according to the following measures: sufficient to cover all living needs and able to save a certain amount, sufficient to cover all living needs, sufficient to cover basic needs only, not sufficient to cover even the basic needs, declined, don’t know. The number of health problems as declared by the respondents ranged from none, 1 to 3, 4 to 6, to more than 7. Alcohol consumption among respondents was assessed in the analysis according to the following categories: non-drinkers, moderate drinkers, and binge drinkers. 

Smoking status. The category of the current tobacco smoking status of the respondents covered current smokers, including daily smokers (smoking one or more cigarettes per day during the past 30 days at a minimum) and occasional smoker (less than daily) subgroups [20,29,30]. Current smokers were also characterized by the age of smoking onset—the age at which the respondents began to smoke tobacco on a regular basis, the number of years of smoking, the number and type of cigarettes consumed per day (regular filter cigarettes, non-filters, hand-rolled, slims, menthols). The intention of the current daily smokers to quit was determined during the study based on the following four categories: intend to quit smoking within the next month; consider quitting smoking within the next 12 months; will quit smoking but not within the next 12 months; do not intend to quit smoking, don’t know/undecided. Respondents who considered quitting smoking within the next 12 months were categorized as interested in quitting smoking, with the rest classified as not interested in quitting. In addition, daily smokers were asked whether they had ever tried to quit (yes/no), to specify the number of quit attempts, their last attempt to quit smoking over the past month, over a period of 1 to 6 months, over a period of 6 to 12 months, and over a period before 12 months. Respondents who declared to be non-smokers were asked if they had ever been daily smokers (yes—former smoker; no—never smoker). The study further assessed the respondents’ awareness of smoking-associated health risks. They were categorized as “aware of the negative health consequences” (positive responses to: “Do you think that tobacco smoking causes serious diseases?”) and “unaware” (negative responses or “don’t know”). Moreover, the study participants were asked if they used e-cigarettes, which was assessed with the following question: “Have you ever (even once) tried e-cigarettes (electronic cigarettes)?” Negative responses were classified as those who “never used e-cigarettes”. Past e-cigarette users were classified as those who had tried e-cigarettes, but had not used them within the month preceding the study. Those who had used e-cigarettes at least once during the past 30 days were classified as current e-cigarette users. Finally, exposure to second hand tobacco smoke was assessed with the use of the following categories: number of hours of daily exposure to tobacco smoke at any place, including home, work, and public places. The total ETS exposure was classified as none, <1 h, 1–5 h, 5–8 h, and more than 8 h per day.

### 2.4. Statistical Analyses

Statistical associations of particular characteristics categories in the analyzed subgroups of respondents were assessed using the chi-square test. All analyses were performed across four age groups: 18–29, 30–39, 40–49, and 50–59 years. The univariable and multivariable logistic regression analysis was implemented to obtain the odds ratios (ORs) and the 95% confidence interval (CI) of each indicator in relation to the current daily smoking of cigarettes. In the first stage, crude coefficients—ORs of the impact of odd variables on the smoking cigarettes in the studied group were calculated. This was followed by a multifactorial analysis considering the simultaneous effect of all variables on the risk of daily smoking. The multiple logistic regression analyses included all variables significantly associated with cigarette use in any of the univariate models (*p* ≤ 0.05). The same scheme was applied in order to calculate the ORs and the 95% CI of each indicator in relation to the intention to quit among the current smokers of cigarettes. All p values were two-sided and *p* <  0.05 was used to indicate the statistical significance. The STATISTICA Windows XP version 10.0 program (StatSoft Poland Inc., Tulusa, OK, USA) was used to complete the statistical analysis.

## 3. Results

### 3.1. Characteristics of the Study Population

In total, 3636 beneficiaries of social assistance were invited to participate. The analysis is based on responses to survey forms received from 1817 SD people (response rate = 49.97%), including 1224 females and 593 males. Table 1 shows the characteristics of the study participants and Table S1 (Supplementary Materials) displays the closest available statistics for all of Poland. The mean age of respondents was 39.2 ± 7.7 years (38.2 ± 7.2 years for females and 41.1 ± 8.1 years for males; *p* < 0.001).

In the studied group, 27.7% of the respondents completed primary education, 32.9% vocational education, 34% secondary education, and 5.4% higher education (Table 1). In relation to females, the majority of the group (almost 40%) completed secondary education, with almost one third reporting vocational training, and almost one fourth only elementary education. Respondents who declared higher education were few, approximately 7% in the female group. For males, the level of education was lower than for females: approximately one third admitted to elementary education only, and almost 40% reported to have completed vocational training. Over 22% of the males completed secondary education and 1.5% received higher education.

The predominant characteristic of the SD population is their low occupational activity: almost 59% of the overall group did not have a job, while only 8.6% were in part-time or temporary employment. Disability or retirement was reported by 3% of the respondents, and student status was declared by 0.1% of the respondents. In the female group, approximately 65% did not have a regular job. Only 25% females acknowledged to having a permanent job, and 6.9% of the females to being in some form of temporary or part-time employment. 2.1% were disabled or retired, and 0.2% of the females were students. Non-working males represented 45% of the group and 38% males acknowledged to having a permanent job, with only 12% of the males being in some form of temporary employment. Almost 5% of the male respondents were disabled or retired.

Just over 1% of the study participants responded that their monthly income was sufficient to cover all their living needs and they could also save a certain amount. Slightly more than 10% of the social assistance beneficiaries declared that their monthly income was sufficient to cover all their living needs, about 52% responded that their monthly income was sufficient to cover the basic needs only, and 25% reported that their income was not sufficient to cover even the basic needs.

About 1.3% of the females responded that their monthly income was sufficient to cover all their living needs and they could also save a certain amount, 11.7% of the females reported that their monthly income was sufficient to cover all their living needs, 53.1% reported that their monthly income was sufficient to cover the basic needs only, and 21.7% reported that their income was not sufficient to cover even the basic needs. For males, 0.7% responded that their monthly income was sufficient to cover all their living needs and they could also save a certain amount, 9.3% reported that their monthly income was sufficient to cover all their living needs, 47.7% responded that their monthly income was sufficient to cover the basic needs only, and 31.7% males responded that their monthly income was not sufficient to cover even the basic needs.

Almost 35% of the respondents assessed their health condition as good, 31% as fairly good, 23.3% as neither fair nor poor, 7.8% as rather poor, and 2.9% as poor. For females, 35.6% assessed their health condition as good, 33.6% as fairly good, 22.6% as neither fair nor poor, 6.6% as rather poor, and 2% as poor. Moreover, 34.3% of the males reported fair health condition, 26.2%—rather fair, 24.7%—neither fair nor poor, 10.4%—rather poor, and 4.9%—poor. Despite the relatively high proportion of positive health assessments, over 80% of the respondents complained of various ailments, and 54.3% declared as many as 1–3 health problems, 26.3% declared 4–6, 5.7%—7 and more health problems simultaneously. For females, 53.8%, 28.7%, 6% reported as many as 1–3, 4–6 and 7 and more health problems respectively. For males, 55.1%, 21.3%, 5.1% reported as many as 1–3, 4–6 and 7 or more health problems respectively (Table 1). 

47% of the respondents declared no drinking at all, while moderate or heavy drinking was declared by about 53% of the social assistance beneficiaries. The data collected on alcohol consumption showed that abstinence was more frequent among females than males (54.9% in females vs. 30.2% in males; *p* < 0.05).

The SD respondents were aware of the damaging effects of smoking. Awareness of smoking-associated health risks was declared by 92.4% of the study participants, including 93.9% females and 89.5% males. Past use of e-cigarettes was reported by 7% of the respondents, current use by 2.3%, with 90.7% of the respondents reporting no previous use of e-cigarettes. The female group included 5.7% of past users, 1.7% of current users, and 92.5% of never users of e-cigarettes. Among men, there were 9.6% past users, 3.5% current users, and 86.8% never users of e-cigarettes. Exposure to environmental tobacco smoke lasting in total less than 1 h per day was declared by 21.6% of the respondents, 9% were exposed from 1–5 h per day, 4.2% were exposed from 5–8 h, and 7.7% were exposed more than 8 h per day. 

### 3.2. Smoking Prevalence and Patterns in the Study Population

Among the 1817 social assistance beneficiaries, 30.8% (*n* = 560) were current daily tobacco smokers, 6.3% (*n* = 115) were current occasional tobacco smokers, 15.3% (*n* = 278) were former tobacco smokers, and 47.6% (*n* = 864) were never smokers (Table 2). Among the female respondents, current daily, current occasional, former and never smokers were 22.1% (*n* = 271); 7.4% (*n* = 91); 14.5% (*n* = 178), and 55.9% (*n* = 684) respectively. The characteristic trait of the population was the high prevalence of smoking, especially among the males (Table 2). Among the 593 male respondents, 48.7% (*n* = 289) were current daily tobacco smokers, 4% (*n* = 24) were current occasional tobacco smokers, 16.9% (*n* = 100) were former tobacco smokers, and 30.4% (*n* = 180) were never smokers. The mean starting age for daily smoking was 19.1 ± 3.9. Women started smoking at a later age than men (19.9 ± 3.96 years vs. 18.2 ± 3.1 years; *p* < 0.0001). 

The mean number of cigarettes smoked per day reported by the current smokers was 14.53 ± 7.23 (Table 2). Daily consumption of cigarettes at 16.17 ± 7.04 per day among men was higher than among the women of 12.89 ± 7.05 cigarettes per day (*p* < 0.0001). Only 4 men and 1 woman smoked tobacco products other than factory-manufactured and/or hand-rolled cigarettes. The mean number of cigarettes smoked daily was 14.4 ± 7.2. The females smoked fewer cigarettes per day than the males (12.9 ± 6.3 vs. 16.2 ± 7.0; *p* < 0.0001).

Out of the 560 current smokers, 74.4% (*n* = 493) declared the use of regular filter cigarettes, 0.8% (*n* = 5) declared non-filters, 14.5% (*n* = 96)—hand-rolled cigarettes, 4.7% (*n* = 31)—slims, and 5.7% (*n* = 38) menthols. Approximately 70% of the females and 80% of the males smoked filter cigarettes (*p* < 0.05), while 12% of the females and 17.5% of the males smoked only hand-rolled cigarettes (*p* < 0.05). The respondents who admitted to smoking only hand-rolled cigarettes smoked more than those reporting smoking filter cigarettes (17.5 ± 8.4 vs. 14.2 ± 6.8; *p* < 0.01) (Table 2). In women, the use of slim and menthol cigarettes was more prevalent than among men (slim users: women 4.7% vs. men 0.6%; *p* < 0.0001, menthol users: women 9.9% vs. men 1%; *p* < 0.0001). Apart from traditional cigarettes, almost 18% of the smokers used electronic cigarettes. Experience of this type of smoking was also reported by the non-smokers: 2.6% of the females and 5.4% of the males (data not presented in the tables).

### 3.3. Intentions to Quit Smoking and Quit Attempts

Just over one third of the smokers reported their willingness to quit smoking, including 16.7% (*n* = 112) of those who intended to quit smoking within the next month, 34.8% (*n* = 234) of those who considered quitting smoking within the next 12 months, 10.1% (*n* = 68) of those who wanted to quit smoking but not within the next 12 months, and 13.4% (*n* = 90) of those who did not intend to quit (Table 3). About one fourth of the respondents did not make any decision in relation to quitting smoking. There were no significant differences between men and women regarding quitting smoking intentions. At the same time, almost 80% of the smokers including 79.6% (*n* = 288) of the females and 78.3% (*n* = 245) of the males tried quitting smoking – 4 times on average. About 15% (*n* = 77) of the respondents tried to quit smoking within the past month, 15.7% (*n* = 83) over a period of 1 month to 6 months, 13.4% (*n* = 71) over a period of 6 to 12 months, and 56.4% (*n* = 299) of the respondents attempted to quit smoking over 12 months prior to the study.

### 3.4. Associates of Current Daily Smoking

The results of the univariate and multivariate regression analyses have been presented in Table 4. Several characteristics, such as the following: male gender, low educational attainment, unemployment or temporary employment, lack of awareness of smoking-associated health risks, use of e-cigarettes and exposure to ETS, were significantly associated with current daily smoking among the beneficiaries of local government welfare assistance from the Piotrkowski district (Table 4). 

### 3.5. Associates of Intentions to Quit

The Odds Ratios (OR) and the 95% Confidence Intervals (CI) for intention to quit smoking within 12 months among daily smokers showed that respondents aware of smoking-associated health risks had much higher odds of actually quitting compared to those unaware of the risks (OR = 6.07; 95% CI: 2.16–17.08; *p* < 0.001). A statistically significant association with the willingness to quit was also found in the respondents who previously attempted to quit compared to those who declared lack of previous quit attempts (OR = 2.81; 95% CI: 1.27–6.25; *p* < 0.001) (Table 5). Moreover, an association between the total daily exposure to ETS (none vs. lower than 1 h per day in total) and the intention to quit was observed for the respondents (OR = 2.14; 95% CI: 1.15–3.96; *p* < 0.05). 

## 4. Discussion

A significantly greater number of men, 33.9% (*n* = 1309) compared to women, 21.9% (*n* = 870) (*p* < 0.01), reported smoking tobacco products on a daily basis (Table 2). These results show a similar pattern to the Poland GATS study [31]. The presented research results also show that smoking prevalence is higher among male beneficiaries of social institutions against those representing the general population [31]. The overall GATS result shows that 30.3% of Poles are current (daily or occasional) smokers; in the study of beneficiaries of social aid 52.8% men and 29.6% of women were current smokers. The results show even deeper discrepancies to the disadvantage of the social aid beneficiaries when they are set against the research covering rural area population in the representative GATS study where 25.4% of rural residents smoked tobacco daily (32.5% men and 17.9% women) [32]. 

According to the presented study, the highest prevalence of smoking was observed in the middle-aged population (aged 40–59). Similar results were shown in the NATPOL 2011 study, as well as in other Polish studies [22,32,33].

In other countries the proportion of smokers among social aid beneficiaries or SD people was also higher compared to the general population. For example, in a research conducted by Bryant et al., daily smoking was reported by 53.5% of the clients of social and community service in Australia compared to the Australian population rate of 15.1% [8]. In other studies concerning various SD subpopulations, smoking rates were also high, e.g., among the low income single women (46%), the homeless (66%–77%) and individuals with a mental illness (41%–62%) [9,10,11,34,35,36,37].

In the study conducted in the Piotrkowski district, the age at smoking onset was no different from the data concerning the general population (18 for men and 20 for women) [38]. In addition, a similar proportion of the participants of the “Your heart—your life” program were never smokers compared to the general population. Daily consumption of cigarettes of 14.5 per day (13 in women and 16 in men) was slightly lower than the general population consumption of 18 cigarettes per day in GATS.

On the other hand, the POLSCREEN study [21] showed consistent results—an average of 16 cigarettes smoked daily by men and 13 by women. Similar values were obtained in other Polish studies [31,33,39]. However, beneficiaries of social aid who smoked hand-rolled cigarettes consumed more than the mean number in the study group (14.5 per day), i.e., over 17 cigarettes per day compared to those who used other types of cigarettes. More effort is required to provide cessation support for this particular group [40,41]. 

Previous quitting attempts were declared by 79% of the respondents from the Piotrkowski district with, on average, four previous quitting attempts (Table 3). This would indicate that many disadvantaged smokers have tried quitting but, unfortunately, have not succeeded in doing so. The present results closely resemble the data from a study by Bryant et al. among social and community service organizations clients in Australia where 77% of the participants tried to quit smoking in the past with an average of two quitting attempts (SD = 3.2) [8]. According to the current data, the proportion of who want to quit is at least as high as the proportion who want to quit in the general population [42]. In this study, 53% of the male and 56% of the female respondents intended to quit smoking, which is in line with the GATS findings. In GATS, more than 50% of the current smokers had an interest in quitting; 31.5% were planning to quit within the next month or were thinking about quitting within the next 12 months, while 18.6% stated they would quit someday but not in the next 12 months. Women (53.1%) were more likely to express a desire to quit than men (48.1%). However, these rates were lower than those observed in several Western countries, where the percentage of smokers who had an interest in quitting smoking ranged from 65% to 81% [43]. The present study revealed that there is some potential for improvement and measures are necessary to increase intentions to quit among disadvantaged smokers, because the intention to quit is the first step towards the goal of quitting smoking completely [44].

Moreover, two important findings were observed based on the present study. The male gender (male vs. female: OR = 2.87; 95% CI: 2.10–3.92; *p* < 0.001) and low level of education (primary vs. higher education: OR = 28.51; 95% CI: 3.71–218.82; *p* < 0.01; vocational vs. higher education: OR = 20.19; 95% CI: 2.63–154.44; *p* < 0.01); secondary vs. higher education: OR = 13.81; 95% CI: 1.80–105.70; *p* < 0.05) were strongly correlated with daily smoking (Table 4). These findings are in line with the GATS results [32]. Both in the rural and urban areas there is a strong difference in cigarette smoking rates based on gender [32]. According to the GATS data, in both the rural and urban areas the lowest percentage of daily cigarette smokers among male respondents was reported for men with higher education [32].

In addition, this study showed that the risk of daily smoking is higher among those who do not receive a regular income than among people with a stable employment, and higher among those who show lack of awareness of smoking-associated health risks (Table 4). This further confirms the results of other studies that have identified a range of factors that influence the uptake and patterns of smoking among high smoking prevalence groups, including: low income, poor housing and unemployment, financial pressure and stress, parental and peer example, nicotine exposure during childhood, targeted and more intensive marketing by the tobacco industry, as well as a lower likelihood of working indoors [45,46,47,48,49]. 

Moreover, a UK study has shown that for every indicator of disadvantage (for example, poor housing, unemployment, single parenthood, etc.) smoking increased by 5% per indicator, up to a total of four indicators. The difference in smoking rates increased at a higher rate between four and five indicators (10%), and by an additional 15% between five and six or more indicators. [8,50].

In the context of the growing expansion of e-cigarettes over the recent years in Poland, it is interesting to note that current daily smoking was associated with past or current e-cigarettes use. The study design does not allow to determine whether this is due to smokers searching for alternative nicotine-containing products to reduce tobacco harm, or due to the use of e-cigarettes as a means to stop smoking traditional cigarettes or, ultimately, due to the so-called dual-use of e-cigarettes and traditional cigarettes. Further research is needed in this area.

The present study has indicated that people with less exposure to ETS had a lower risk of daily smoking, but did not directly give information on this subject. However, the lack of exposure may be a consequence of smoking bans in the workplace, which prompts quitting attempts and reductions in consumption. Moreover, the majority of non-smokers at home or at work perceive tobacco as less acceptable [51,52,53].

It should be also underlined that the most important factor correlated with an intention to quit was the awareness of smoking-associated health risks (Table 5). The finding in the current study is consistent with earlier studies conducted in other countries [44,54,55]. For instance, Driezen at al. in a study from Bangladesh found that smokers’ concerns about the health risks significantly influenced their odds of planning to quit: smokers who were very worried about their health had close to 9 times higher odds of planning to quit compared to smokers who were not worried at all [44]. Findings from the International Tobacco Control Bangladesh have revealed that smokers who were moderately concerned had over 4 times higher odds of planning to quit, while smokers who were only a little worried about their health had 3.9 times higher odds of planning to quit [44]. Additional efforts are required from health policy-makers and health care professionals to disseminate information about the risks of smoking and the health benefits of quitting.

Contrary to the findings of a previous study by Driezen et al, the current study found that the respondents with no previous quitting attempts were more likely to report an intention to quit smoking within 12 months [44]. Previous research has shown that past quitting efforts were associated with quitting intentions compared to making no effort [44]. Hypothetically, these findings are likely to be due to the fact that no success in smoking cessation may result in smokers losing their motivation to quit, and therefore, having no further intention to quit. [56,57]. Unfortunately, the study did not address motivations or beliefs of the disadvantaged smokers concerning smoking and quitting that could serve as barriers to quitting. Little is known about the smoking and quitting beliefs of the very poor in Poland. Existing research indicates that when low socioeconomic smokers do try to quit, they are less likely to be successful than other smokers [8,56]. This lack of cessation success may reflect, to some extent, no use of evidence-based smoking cessation treatment methods like, for instance, cessation medication, counseling or behavioral interventions [58,59,60]. Christiansen et al. suggest that efforts to increase quitting attempts and to engage deprived smokers in treatment may have to address specific beliefs about smoking and quitting [57]. Some authors additionally suggest the need to motivate smokers to think about quitting by raising their awareness of the benefits of quitting smoking through educational campaigns [61].

It should also be taken into consideration that the local community agencies may be an important setting and a partner in reducing smoking rates among the disadvantaged. They have the opportunity to make a great impact because of their proximity to the target population [8,57,61].

Bringing cessation services to the disadvantaged smokers in familiar environments has been recommended as a vital strategy to increase utilization of cessation methods [62,63].

There is a growing body of research exploring internationally the role of these services in tobacco control. Surveys of smokers who are clients of community services have indicated they are open to receiving smoking cessation assistance from these services as they are a trusted source of advice and support and, therefore, can offer more personalized support [62,63,64,65].

Integration of smoking cessation support in organizations that already work with the disadvantaged groups has been shown to be effective in decreasing smoking rates in several countries. 

However, social assistance institutions in Poland do not take part in anti-tobacco activities, and when they do, they do so to a little extent only. There is a lack of research in the field of the interest in this type of assistance among social and community service clients. Further, there are no data concerning the preparation of the social and community service institution staff to join anti-tobacco programs. 

## 5. Study Limitations and Strength

Study limitations include questionnaire based assessment of smoking patterns and associated potential recall bias, personal perception and the use of a cross-sectional study. The limitation of the recall bias is evident in the self-reporting process of data collection. There are other methods that can be included in the survey data collection. For example, biomarkers of tobacco use such as cotinine or carbon monoxide could be included, but this would generate excessive costs and take more time—this is the reason for their infrequent use in large epidemiological surveys [66]. Cross-sectional surveys tend to make observations at a single point in time. This, in turn, leads to another study limitation, which is the inability to observe the analyzed associations or determinants among the disadvantaged population over their lifetime. In addition, no data on the mental health history of the respondents are available. The mental health condition is a strong predictor of smoking and may be associated with smoking cessation intentions and quitting success [34,67,68]. The current analysis has several strengths. For the first time the study was conducted among a socially disadvantaged population of social assistance beneficiaries from rural district of Poland. The fact that all the beneficiaries of social aid aged 18–59 were invited to participate in the study, and a half of the invited group participated in the survey, constitutes another significant advantage of the current assessment and a guarantee of valid data for the studied subpopulation. The response rate in the present study was 50% of the average level compared to other questionnaire surveys in Poland. For instance, the overall survey participation rate was over 60% in the GATS Poland, with the response rate of 45% [19,20] in the Multi-Centre National Population Health Examination Survey (WOBASZ II study). Finally, interviewer-administered questionnaires produce higher values of sensitivity and specificity compared to self-administered questionnaires, and helped to reduce the non-response rate [66]. Despite some limitations, the current study provides representative data and a valuable insight into the prevalence, patterns and correlates of cigarette use and willingness to quit among social aid recipients.

## 6. Conclusions

This is probably the first study to explore smoking patterns and intentions to quit among the SD population receiving aid from community service organizations in the area of welfare support in Poland. There is a need to develop comprehensive policies tailored to the different needs of such disadvantaged population groups [13,69]. The findings have identified multiple factors associated with the daily smoking and the intention to quit. In line with the research completed to date in Poland, the general conclusion could be that the prevalence of smoking in rural communities is lower compared to urban areas, the socioeconomic situation has a significant impact on some rural communities, while lower incomes, higher unemployment rates and lower education levels contribute to higher smoking prevalence among rural populations, recipients of social aid. At the same time, many smokers who receive social aid intend to quit. As such, targeted and effective strategies must be developed which should take into account the fact that the risk of daily smoking is very much correlated with the level of education, which should become a cue for strengthening anti-tobacco campaigns among school students—elementary education is prevalent among people with lower incomes, being only incidental with higher education. The fact that the risk of daily smoking is higher among the smokers who are not aware of the health risks than among those who are aware of the dangers, as well as the fact that being aware of the negative effects of smoking helps the intention to quit should become an incentive for enhanced anti-tobacco campaigns, including media and educational campaigns focusing on the risks of tobacco use [70]. Improving access to cessation services and treatments in high smoking prevalence groups may be the promising approach for reducing smoking rates. The strengthening of smoke-free legislation, its enforcement and compliance may discourage smokers from smoking and help nonsmokers and quitters to maintain abstinence [71,72]. Other interventions, for instance price and tax regulations that lead to decreased use of hand-rolled cigarettes as a result of limited affordability of loose tobacco used for preparing hand-rolled cigarettes should also be considered [13]. Interventions and programs could potentially be delivered by organizations offering assistance to the disadvantaged groups, but further research is required to explore this area.

## Figures and Tables

**Table 1:** Characteristics of study respondents *n* = 1817.

Variable	Total Sample	Women				Men			
		Total	Smokers	Non-Smokers	*p*	Total	Smokers	Non-Smokers	*p*
Overall	*n* = 1817	*n* = 1224	*n* = 362 (29.6%)	*n* = 862 (70.4%)		*n* = 593	*n* = 313 (52.8%)	*n* = 280 (47.2%)	
	*n* (%)	*n* (%)	*n* (%)	*n* (%)		*n* (%)	*n* (%)	*n* (%)	
**Age (years)**									
18–29	209 (11.5)	160 (76.6)	45 (28.1)	115 (71.9)	*p* > 0.05	49 (23.4)	30 (61.2)	19 (38.8)	*p* > 0.05
30–39	778 (42.8)	566 (72.8)	151 (26.7)	415 (73.3)		212 (27.2)	102 (48.1)	110 (51.9)	
40–49	601 (33.1)	385 (64.1)	129 (33.5)	256 (66.5)		216 (35.9)	111 (51.4)	105 (48.6)	
50–59	229 (12.6)	113 (49.3)	37 (32.7)	76 (67.3)		116 (50.7)	70 (60.3)	46 (39.7)	
**Education**									
primary	493 (27.7)	283 (57.4)	113 (39.9)	170 (60.1)	*p* < 0.0001	210 (42.6)	137 (65.2)	73 (34.8)	*p* < 0.0001
vocational	586 (32.9)	351 (59.9)	113 (32.2)	238 (67.8)		235 (40.1)	128 (54.5)	107 (45.5)	
secondary	606 (34.0)	475 (78.4)	122 (25.7)	353 (74.3)		131 (21.6)	43 (32.8)	88 (67.2)	
high	96 (5.4)	88 (91.7)	7 (8.0)	81 (92.0)		8 (8.3)	1 (12.5)	7 (87.5)	
Missing data	36 (2.0)	27 (2.2)	7 (1.9)	20 (2.3)		9 (1.5)	4 (1.3)	5 (1.8)	
**Employment status**									
permanent job	533 (29.5)	310 (58.2)	79 (25.5)	231 (74.5)	*p* > 0.05	223 (41.8)	82 (36.8)	141 (63.2)	*p* < 0.0001
temporary job	156 (8.6)	85 (54.5)	34 (40.0)	51 (60.0)		71 (45.5)	47 (66.2)	24 (33.8)	
disabled or retired	54 (3.0)	26 (48.1)	6 (23.1)	20 (76.9)		28 (51.9)	15 (53.6)	13 (46.4)	
student	2 (0.1)	2 (100.0)	1 (50.0)	1 (50.0)		0	0	0	
unemployed	1060 (58.7)	793 (74.8)	241 (30.4)	552 (69.6)		267 (25.2)	167 (62.5)	100 (37.5)	
Missing data	12 (0.7)	8 (0.7)	1 (0.3)	7 (0.8)		4 (0.7)	2 (0.6)	2 (0.7)	
**Subjective assessment of monthly income**									
sufficient to cover all living needs plus may save a certain amount	20 (1.1)	16 (80.0)	2 (12.5)	14 (87.5)	*p* < 0.0001	4 (20.0)	2 (50.0)	2 (50.0)	*p* < 0.0001
sufficient to cover all living needs	198 (10.9)	143 (72.2)	26 (18.2)	117 (81.8)		55 (27.8)	14 (25.5)	41 (74.5)	
sufficient to cover basic needs only	933 (51.5)	650 (69.7)	193 (29.7)	457 (70.3)		283 (30.3)	142 (50.2)	141 (49.8)	
not sufficient to cover even the basic needs	454 (25.1)	266 (58.6)	101 (38.0)	165 (62.0)		188 (41.4)	125 (66.5)	63 (33.5)	
Declined response	64 (3.5)	42 (65.6)	7 (16.7)	35 (83.3)		22 (34.4)	8 (36.4)	14 (63.6)	
difficult to say	141 (7.8)	101 (71.6)	32 (31.7)	69 (68.3)		40 (28.4)	21 (52.5)	19 (47.5)	
Missing data	7 (0.4)	6 (0.5)	1 (0.3)	5 (0.6)		1 (0.2)	1 (0.3)	0 (0.0)	
**Subjective health state**									
fair	632 (35.0)	434 (35.6)	112 (25.8)	322 (74.2)	*p* > 0.05	198 (34.3)	107 (54.0)	91 (46.0)	*p* > 0.05
rather fair	559 (31.0)	405 (33.6)	121 (29.9)	284 (60.1)		154 (26.2)	77 (50.0)	77 (50.0)	
neither fair nor poor	420 (23.3)	275 (22.6)	89 (32.4)	186 (67.6)		145 (24.7)	78 (53.8)	67 (46.2)	
rather poor	141 (7.8)	80 (6.6)	31 (38.7)	49 (61.3)		61 (10.4)	30 (49.2)	31 (50.8)	
poor	53 (2.9)	24 (2.0)	7 (29.2)	17 (60.8)		29 (4.9)	20 (69.0)	9 (31.0)	
Missing data	12 (0.7)	6 (0.5)	2 (0.6)	4 (0.5)		6 (1.0)	1 (0.3)	5 (1.8)	
**Number of health problems**									
none	245 (13.7)	137 (11.4)	35 (25.5)	102 (74.5)	*p* > 0.05	108 (18.4)	61 (56.5)	47 (43.5)	*p* > 0.05
1–3 health problems	968 (54.3)	645 (53.8)	176 (27.3)	469 (72.7)		323 (55.1)	176 (54.5)	147 (45.5)	
4–6 health problems	469 (26.3)	344 (28.7)	115 (33.4)	229 (66.6)		125 (21.3)	58 (46.4)	67 (53.6)	
≥ 7 health problems	102 (5.7)	72 (6.0)	24 (33.3)	48 (66.7)		30 (5.1)	13 (43.3)	17 (56.7)	
Missing data	33 (1.8)	26 (2.1)	12 (2.1)	14 (1.6)		7 (1.2)	5 (1.6)	2 (0.7)	
**Alcohol consumption**									
do not drink at all	803 (47.0)	638 (54.9)	170 (26.4)	468 (73.6)	*p* < 0.05	165 (30.2)	74 (44.8)	91 (55.2)	*p* < 0.05
moderate drinking and heavy drinking	903 (52.9)	524 (45.1)	170 (32.4)	354 (67.6)		379 (69.4)	206 (54.4)	173 (45.6)	
Missing data	109 (6.0)	62 (5.1)	22 (6.1)	40 (4.6)		47 (7.9)	31 (9.9)	16 (5.7)	
**Awareness of smoking-associated health risks**									
yes	1677 (92.4)	1147 (93.9)	320 (27.9)	827 (72.1)	*p* < 0.0001	530 (89.5)	274 (51.7)	256(48.3)	*p* > 0.05
no	137 (7.6)	75 (6.1)	41 (54.7)	34 (35.3)		62 (10.5)	39 (62.9)	23 (37.1)	
Missing data	3 (0.2)	2 (0.2)	1 (0.3)	1 (0.1)		1 (0.2)	0 (0.0)	1 (0.4)	
**Use of e-cigarettes**									
past use	127 (7.0)	70 (5.7)	53 (75.7)	17 (24.3)	*p* < 0.0001	57 (9.6)	47 (82.5)	10 (17.5)	*p* < 0.0001
current use	42 (2.3)	21 (1.7)	16 (76.2)	5 (23.8)		21 (3.5)	16 (76.2)	5 (23.8)	
never use	1642 (90.7)	1129 (92.5)	292 (25.9)	837 (74.1)		513 (86.8)	250 (48.7)	263 (51.3)	
Missing data	6 (0.3)	4 (0.3)	1 (0.3)	3 (0.4)		2 (0.3)	0 (0.0)	2 (0.3)	
**ETS exposure (total number of hours per day)**									
0	1041 (57.6)	744 (61.1)	149 (20.0)	595 (80.0)	*p* < 0.0001	297 (50.2)	100 (33.7)	197 (66.3)	*p* < 0.0001
<1h	390 (21.6)	268 (22.0)	109 (40.7)	159 (59.3)		122 (20.4)	70 (57.4)	52 (42.6)	
1–5h	162 (9.0)	90 (7.4)	32 (35.6)	58 (64.4)		72 (12.2)	55 (76.4)	17 (23.6)	
5–8h	76 (4.2)	44 (3.6)	21 (47.7)	23 (52.3)		32 (5.4)	22 (68.8)	10 (31.3)	
More than 8h	140 (7.7)	72 (5.9)	48 (66.7)	24 (33.3)		68 (11.5)	64 (94.1)	4 (5.9)	
Missing data	8 (0.4)	6 (0.5)	3 (0.8)	3 (0.3)		2 (0.3)	2 (0.6)	0 (0.0)	

ETS: Environmental Tobacco Smoke.

**Table 2:** Smoking characteristic of the study sample *n* = 1817.

Smoking Status	Total *n*	%	Women		Men		*p*
			*n*	% (95% CI)	*n*	% (95% CI)	
Current daily smoker	560	30.8	271	22.1 (19.8–24.4)	289	48.7 (44.7–52.7)	*p* < 0.0001
Current occasional smoker	115	6.3	91	7.4 (5.9–8.9)	24	4.0 (2.4–5.6)	*p* < 0.05
Never smoker	864	47.6	684	55.9 (53.1–58.7)	180	30.4 (26.7–34.1)	*p* < 0.0001
Former smoker	278	15.3	178	14.5 (12.5–16.5)	100	16.9 (13.9–19.9)	*p* > 0.05
Missing data	1	0.06	1	0.08	0		
			The type of cigarettes smoked the most				
Regular filter cigarettes	493	74.4	246	69.5 (64.7–74.3)	247	79.9 (75.4–84.4)	*p* < 0.05
Non-filter	5	0.8	2	0.6 (0.0–1.4)	3	1.0 (0.0–2.1)	*p* > 0.05
Hand-rolled	96	14.5	42	11.9 (8.5–15.3)	54	17.5 (13.3–21.7)	*p* < 0.05
Slim	31	4.7	29	8.2 (5.3–11.1)	2	0.6 (0.0–1.5)	*p* < 0.0001
Menthol	38	5.7	35	9.9 (6.8–13.0)	3	1.0 (0.0–2.1)	*p* < 0.0001
Missing data	12	1.8	8	2.2	4 1.3		
Age at smoking onset Mean (± SD)		19.10 ± 3.96	19.88 ± 4.44		18.18 ± 3.08		*p* < 0.0001
Number of cigarettes smoked daily Mean (± SD)		14.53 ± 7.23	12.89 ± 7.05		16.17 ± 7.04		*p* < 0.0001
Filter		14.18 ± 6.86	12.60 ± 6.27		15.63 ± 7.06		*p* < 0.0001
Non-filter		15.00 ± 5.00	10.00		17.50 ± 3.54		*p* > 0.05
Hand-rolled		17.48 ± 8.40	16.71 ± 10.42		18.00 ± 6.75		*p* > 0.05
Slim		12.59 ± 6.08	11.85 ± 5.88		20.00 ± 0.00		-
Menthol		10.89 ± 7.23	9.82 ± 5.89		20.00 ± 14.14		-

SD: standard deviation.

**Table 3:** Quitting intentions and attempts among the daily and occasional smokers sample.

Quitting Intentions			Total *n*	Total %	Women		Men		*p*
					*n*	% (95% CI)	*n*	% (95% CI)	
Intend to quit smoking within the next month			112	16.7	63	17.5 (13.6–21.4)	49	15.8 (11.7–19.8)	*p* > 0.05
Consider quitting smoking within the next 12 months			234	34.8	129	35.7 (30.8–40.6)	105	33.8 (28.6–39.1)	*p* > 0.05
Will quit smoking but not within the next 12 months			68	10.1	37	10.2 (7.1–13.3)	31	10.0 (6.7–13.3)	*p* > 0.05
Do not intend to quit smoking			90	13.4	43	11.9 (8.6–15.2)	47	15.1 (11.1–19.1)	*p* > 0.05
Don’t know/undecided			168	25.0	89	24.6 (20.1–29.0)	79	25.4 (20.6–30.2)	*p* > 0.05
Missing data			3	0.4	1	0.3	2	0.6	
			**Previous quit attempts**						
No			131	19.4	69	19.1 (15.0–23.2)	62	19.8 (15.3–24.3)	*p* > 0.05
Yes			533	79.0	288	79.6 (75.4–83.8)	245	78.3 (73.7–82.9)	*p* > 0.05
Missing data			11	1.6	5	1.4	6	1.9	
Number of previous quit attempts Mean (±SD)				3.49 ± 3.32	3.71 ± 3.76		3.21 ± 2.69		*p* > 0.05
			**Time of the last attempt to quit smoking**						
Within the last month			77	14.5	51	17.8 (13.9–1.7)	26	10.7 (7.3–14.1)	*p* < 0.01
1 to 6 months			83	15.7	52	18.1 (14.1–22.1)	31	12.8 (9.1–16.5)	*p* > 0.05
6 to 12 months			71	13.4	42	14.6 (11.0–18.2)	29	11.9 (8.3–15.5)	*p* > 0.05
Over 12 months prior to study			299	56.4	142	49.5 (44.3–54.7)	157	64.6 (59.3–69.9)	*p* < 0.0001
Missing data			3	0.6	1	0.3	2	0.8	

SD: standard deviation.

**Table 4:** Odds Ratios (OR) and 95% Confidence Intervals (CI) for daily smoking to selected socio-demographic characteristics in adult men and women from the Piotrkowski district.

Variable	Total (*n*)	Daily Smokers		Univariate Logistic Regression		Multivariate Logistic Regression ^a^	
		*n*	%	OR	95% CI	OR	95% CI
**Gender**							
male	366	173	47.3	3.19 ***	2.45–4.16	2.87 ***	2.10–3.92
female	802	176	21.9	1.00	reference	1.00	reference
**Age (years)**							
18–29	122	36	29.5	1.00	reference	1.00	reference
30–39	508	129	25.4	0.81	0.52–1.26		
40–49	395	137	34.7	1.27	0.82–1.97		
50–59	143	47	32.9	1.17	0.69–1.97		
**Education**							
primary	324	135	41.7	40.71 ***	5.49–301.85	28.51 **	3.71–218.82
vocational	399	130	32.6	27.55 ***	3.73–203.42	20.19 **	2.63–154.44
secondary	387	83	21.5	15.56 **	2.11–115.04	13.81 *	1.80–105.70
high	58	1	1.7	1.00	Reference	1.00	reference
**Occupational classification**							
permanent job	344	88	25.6	1.00	Reference	1.00	reference
temporary job	94	41	43.6	2.25 ***	1.40–3.62	1.84 *	1.05–3.22
economically not active (disabled or retired)	32	5	15.6	0.54	0.20–1.44	0.40	0.13–1.23
unemployed	698	215	30.8	1.29	0.97–173	1.58 *	1.11–2.25
**Subjective assessment of monthly income**							
sufficient to cover all living needs plus may save a certain amount	14	4	28.6	1.00	reference	1.00	reference
sufficient to cover all living needs	130	20	15.4	0.45	0.13–1.60		
sufficient to cover basic needs only	635	195	30.7	1.11	0.34–3.58		
not sufficient to cover even the basic needs	289	112	38.7	1.58	0.48–5.17		
difficult to say/decline to answer	100	18	18.0	0.55	0.15–1.95		
**Subjective health state**							
fair	415	118	28.4	1.00	reference	1.00	reference
rather fair	352	99	28.1	0.98	0.72–1.35		
neither fair nor poor	274	87	31.7	1.17	0.84–1.63		
rather poor	95	30	31.6	1.16	0.72–1.88		
poor	32	15	46.7	2.22*	1.07–4.59		
**Number of health problems**							
none	157	39	24.8	1.00	reference	1.00	reference
1–3 health problems	621	195	31.4	1.38	0.93–2.07		
4–6 health problems	319	93	29.2	1.25	0.81–1.92		
≥7 health problems	71	22	31.0	1.36	0.73–2.53		
**Alcohol consumption**							
do not drink at all	540	131	24.3	1.00	reference	1.00	reference
moderate and heavy drinking	628	218	34.7	1.66 ***	1.28–2.15		
**Awareness of smoking-associated health risks**							
yes	1105	318	28.8	1.00	reference	1.00	reference
no	63	31	49.2	2.40 ***	1.44–4.00	1.97 *	1.10–3.56
**Use of e-cigarettes**							
past use	72	50	69.4	6.37 ***	3.78–10.71	8.16 ***	4.50–14.78
current use	28	18	64.3	5.04 ***	2.30–11.07	3.47 **	1.39–8.64
never use	1068	281	26.3	1.00	reference	1.00	reference
**ETS exposure** number of hours per day							
0	646	121	18.7	1.00	reference	1.00	reference
<1 h	274	98	35.8	2.42 ***	1.76–3.32	2.29 ***	1.61–3.25
1–5 h	119	50	42.0	3.14 ***	2.08–4.76	2.50 ***	1.58–3.94
5–8 h	56	29	51.8	4.66 ***	2.66–8.17	3.98 ***	2.16–7.35
>8 h	73	51	69.9	10.06 ***	5.87–17.24	8.02 ***	4.46–14.42

^a^ Fully adjusted model including all statistically significant characteristics. ETS—environmental tobacco smoke. *** *p* ≤ 0.001; ** *p* ≤ 0.01; * *p* ≤ 0.05.

**Table 5:** Odds Ratios (OR) and 95% Confidence Intervals (CI) for the intention to quit smoking within 12 months to selected socio-demographic characteristics in men and women - current daily smoking from the Piotrkowski district.

Variable	Total (*n*)	Daily Smokers Intention to Quit Smoking within 12 Months		Univariate Logistic Regression		Multivariate Logistic Regression ^a^	
		*n*	%	OR	95% CI	OR	95% CI
**Gender**							
male	144	77	53.5	1.00	reference	1.00	reference
female	152	85	55.9	1.10	0.70–1.75		
**Age (years)**							
18–29	29	15	51.7	1.00	reference	1.00	reference
30–39	110	53	48.2	0.87	0.38–1.97		
40–49	120	69	57.5	1.26	0.56–2.86		
50–59	37	25	67.6	1.94	0.71–5.32		
**Age at smoking onset (years)**							
≥17	89	50	56.2	0.73	0.35–1.51		
18–20	160	82	51.2	0.60	0.30–1.16		
>20	47	30	63.8	1.00	reference	1.00	reference
**Education**							
primary	110	63	57.3	1.17	0.65–2.12	1.00	reference
vocational	111	59	53.2	0.99	0.55–1.79		
secondary	75	40	53.3	1.00	reference	1.00	reference
high	0						
**Occupational classification**							
permanent job	78	42	53.8	1.00	Reference	1.00	reference
temporary job	39	19	48.7	0.81	0.38–1.76		
economically not active (disabled or retired)	3	2	66.7	1.71	0.15–19.91		
unemployed	176	99	56.3	1.10	0.64–1.89		
**Subjective assessment of monthly income**							
sufficient to cover all living needs plus may save a certain amount	3	1	33.3	1.00	reference	1.00	reference
sufficient to cover all living needs	19	8	42.1	1.45	0.11–19.12		
sufficient to cover basic needs only	168	97	57.7	2.73	0.24–31.08		
not sufficient to cover even the basic needs	89	50	56.2	2.56	0.22–29.66		
difficult to say/decline to answer	17	6	35.3	1.09	0.08–14.83		
**Subjective health state**							
fair	102	58	58.9	1.00	reference	1.00	reference
rather fair	87	50	57.5	1.03	0.57–1.83		
neither fair nor poor	71	33	46.5	0.66	0.36–1.21		
rather poor	24	16	66.7	1.52	0.59–3.88		
poor	12	5	41.7	0.54	0.16–1.83		
**Number of health problems**							
none	35	23	65.7	1.00	reference	1.00	reference
1–3 health problems	165	82	49.7	0.52	0.24–1.11		
4–6 health problems	77	46	59.7	0.77	0.34–1.79		
≥7 health problems	19	11	57.9	0.72	0.23–2.27		
**Alcohol consumption**							
do not drink	109	62	56.9	1.00	reference	1.00	reference
moderate and heavy	187	100	53.5	0.87	0.54–1.40		
**Awareness of smoking-associated health risks**							
yes	270	157	58.2	5.84 ***	2.12–16.03	6.07 ***	2.16-17.08
no	26	5	19.2	1.00	reference	1.00	reference
**Number of cigarettes smoked per day**							
<10	64	44	68.7	2.20 *	1.13–4.30		
10–20	138	71	51.5	1.06	0.63–1.79		
>20	94	47	50.0	1.00	reference	1.00	reference
**Use of e-cigarettes**							
past use	45	26	57.8	1.14	0.60–2.17		
current use	11	5	45.5	0.69	0.20–2.35		
never use	240	131	54.6	1.00	reference	1.00	reference
**ETS exposure (total number of hours per day)**							
0	104	49	47.1	1.00	reference	1.00	reference
<1 h	85	55	64.7	2.06 *	1.14–3.72	2.14 *	1.15–3.96
1–5 h	42	27	64.3	2.02	0.96–4.25	2.14 ^#^	0.99–4.66
5–8 h	25	12	48.0	1.04	0.43–2.49	1.12	0.45–2.78
>8 h	40	19	47.5	1.02	0.49–2.11	1.04	0.49–2.24
**Previous quit attempts**							
yes	34	11	32.4	1.00	reference	1.00	reference
no	262	151	57.6	2.84 **	1.33–6.10	2.81 **	1.27–6.25

^a^ Fully adjusted model including statistically significant characteristics. *** *p* ≤ 0.001; ** *p* ≤ 0.01; * *p* ≤ 0.05. ^#^ *p* = 0.055.

## Supplementary Materials

The following are available online at http://www.mdpi.com/1660-4601/14/2/131/s1, Table S1, Distribution of adults ≥15 years old by selected demographic characteristics—GATS Poland, 2009–2010.

## References

[1] Eriksen M., Mackay J., Schluger N., Gomeshtapeh F.I., Drope J. (2015). The Tobacco Atlas.

[2] Eriksen M., Mackay J., Ross H. (2012). The Tobacco Atlas.

[3] World Health Organization (2012). WHO Global Report: Mortality Attributable to Tobacco.

[4] Jha P., Peto R., Zatonski W., Boreham J., Jarvis M.J., Lopez A.D. (2006). Social inequalities in male mortality, and in male mortality from smoking: Indirect estimation from national death rates in England and Wales, Poland, and North America. Lancet.

[5] World Health Organization (2014). Tobacco and Inequalities. Guidance for Addressing Inequities in Tobacco-Related Harm.

[6] Hiscock R., Bauld L., Amos A., Platt S. (2012). Smoking and socioeconomic status in England: The rise of the never smoker and the disadvantaged smoker. J. Public Health (Oxf.).

[7] Tsai J., Rosenheck R.A. (2012). Smoking among chronically homeless adults: Prevalence and correlates. Psychiatr. Serv..

[8] Bryant J., Bonevski B., Paul C. (2011). A survey of smoking prevalence and interest in quitting among social and community service organisation clients in Australia: A unique opportunity for reaching the disadvantaged. BMC Public Health.

[9] Siahpush M., Borland R., Scollo M. (2002). Health Inequalities: Prevalence and socio-economic correlates of smoking among lone mothers in Australia. Aust. N. Z. J. Public Health.

[10] Johnson T.P., Barrett M.E. (1995). Substance use and treatment needs among homeless persons in Cook County, Illinois. Int. J. Addict..

[11] Connor S.E., Cook R.L., Herbert M.I., Neal S.M., Williams J.T. (2002). Smoking cessation in a homeless population: There is a will, but is there a way?. J. Gen. Intern. Med..

[12] Cavelaars A.E., Kunst A.E., Geurts J.J., Crialesi R., Grötvedt L., Helmert U., Lahelma E., Lundberg O., Matheson J., Mielck A. (2000). Educational differences in smoking: International comparison. BMJ.

[13] World Health Organization (2009). WHO Report on the Global Tobacco Epidemic, 2008—The MPOWER Package.

[14] Pampel F.C., Krueger P.M., Denney J.T. (2010). Socioeconomic Disparities in Health. Behav. Annu. Rev. Sociol..

[15] World Health Organisation (2008). MPOWER: Six Policies to Reverse the Tobacco Epidemic.

[16] World Health Organisation (2003). Framework Convention on Tobacco Control.

[17] Bonevski B., Randell M., Paul C., Chapman K., Twyman L., Bryant J., Brozek I., Hughes C. (2014). Reaching the hard-to-reach: A systematic review of strategies for improving health and medical research with socially disadvantaged groups. BMC Med. Res. Methodol..

[18] Depa J., Hilzendegen C., Tinnemann P., Stroebele-Benschop N. (2015). An explorative cross-sectional study examining self-reported health and nutritional status of disadvantaged people using food banks in Germany. Int. J. Equity Health.

[19] Kaleta D., Usidame B., Biliński P., Racibrski F., Samoliński B., Wojtyła A., Fronczak A. (2012). Global Adult Tobacco Survey (GATS) in Poland 2009–2010 study strengths, limitations and lessons learned. Ann. Agric. Environ. Med..

[20] Drygas W., Niklas A.A., Piwońska A., Piotrowski W., Flotyńska A., Kwaśniewska M., Nadrowski P., Puch-Walczak A., Szafraniec K., Bielecki W. (2016). Multi-centre National Population Health Examination Survey (WOBASZ II study): Assumptions, methods, and implementation. Kardiol. Pol..

[21] Podolec P., Kopeć G., Cieśliński A. (2006). Rozpowszechnienie palenia tytoniu wśród dorosłych Polaków—Wyniki badania POLSCREEN. Ogólnopolski Program Prewencji Choroby Wieńcowej POLSCREEN.

[22] Kopeć G., Jankowski P., Pająk A., Drygas W. (2015). Epidemiology and Prevention of Cardiovascular Diseases.

[23] Zdrojewski T., Rutkowski M., Bandosz P., Gaciong Z., Jędrzejczyk T., Solnica B., Pencina M., Drygas W., Wojtyniak B., Grodzicki T. (2013). Prevalence and control of cardiovascular risk factors in Poland. Assumptions and objectives of the NATPOL 2011 Survey. Kardiol. Pol..

[24] Central Statistical Office (2015). Beneficiaries of Social Assistance and Family Benefits in 2014.

[25] Assessment of Health Needs of the Residents of Piotrkowski District 2014. http://www.zdrowie.powiatpiotrkowskipl/download/Download/Ocena_potrzeb_zdrowotnych_powiat_piotrkowski.pdf.

[26] United Nations Development Programme, National Human Development Report. Regional and Local Development. http://issuu.com/undp_poland/docs/lhdi_report_poland_2012_eng.

[27] World Health Organization (2012). Social Inequalities in Health in Poland.

[28] Kaleta D., Wojtysiak P., Polańska K. (2016). Use of electronic cigarettes among secondary and high school students from a socially disadvantaged rural area in Poland. BMC Public Health.

[29] Polańska K., Wojtysiak P., Bąk-Romaniszyn L., Kaleta D. (2016). Susceptibility to cigarette smoking among secondary and high school students from a socially disadvantaged rural area in Poland. Tob. Induc. Dis..

[30] Pikala M., Kaleta D., Bielecki W., Maniecka-Bryła I., Drygas W., Kwaśniewska M. (2011). Awareness of cardiovascular prevention methods among residents of post-communist Polish provinces with highest mortality rates. Cent. Eur. J. Public Health.

[31] Kaleta D., Makowiec-Dąbrowska T., Dziankowska-Zahorszczyk E., Fronczak A. (2012). Prevalence and sociodemographic correlates of daily cigarette smoking in Poland: Results from the Global Adult Tobacco Survey (2009–2010). Int. J. Occup. Med. Environ. Health.

[32] Włodarczyk A., Raciborski F., Opoczyńska D., Samoliński B., GATS PWG (2013). Daily tobacco smoking patterns in rural and urban areas of Poland—The results of the GATS study. Ann. Agric. Environ. Med..

[33] Jóźwiak P., Wierzejska E., Szmagaj A., Biskupska M. (2014). Risk factors for smoking in persons over 45. Przeglad Lekarski.

[34] De Leon J., Diaz F.J. (2005). A meta-analysis of worldwide studies demonstrates an association between schizophrenia and tobacco smoking behaviors. Schizophr. Res..

[35] Lasser K., Boyd J.W., Woolhandler S., Himmelstein D.U., McCormick D., Bor D. (2000). Smoking and mental illness: A population-based prevalence study. JAMA.

[36] Baggett T.P., Rigotti N.A. (2010). Cigarette smoking and advice to quit in a national sample of homeless adults. Am. J. Prev. Med..

[37] Kermode M., Crofts N., Miller P., Speed B., Streeton J. (1998). Health indicators and risks among people experiencing homelessness in Melbourne, 1995–1996. Aust. N. Z. J. Public Health.

[38] World Health Organization (2010). GATS Country Report. Global Adult Tobacco Survey Poland 2009–2010.

[39] Czapiński J., Panek T. (2013). Diagnoza Społeczna 2013, Warunki I Jakość Życia Polaków—Społecznego.

[40] Bryant J., Bonevski B., Paul C., O’Brien J., Oakes W. (2011). Developing cessation interventions for the social and community service setting: A qualitative study of barriers to quitting among disadvantaged Australian smokers. BMC Public Health.

[41] Murray R., Bauld L., Hacksha L., McNeill A. (2009). Improving access to smoking cessation services for disadvantaged groups: A systematic review. J. Public Health.

[42] Lebrun-Harris L.A., Fiore M.C., Tomoyasu N., Ngo-Metzger Q. (2015). Cigarette smoking, desire to quit, and tobacco-related counseling among patients at adult health centers. Am. J. Public Health.

[43] Hyland A., Laux F.L., Higbee C., Hastings G., Ross H., Chaloupka F.J., Fong G.T., Cummings K.M. (2006). Cigarette purchase patterns in four countries and the relationship with cessation: Findings from the International Tobacco Control (ITC) Four Country Survey. Tob. Control.

[44] Driezen P., Abdullah A.S., Quah A.C., Nargis N., Fong G.T. (2016). Determinants of intentions to quit smoking among adult smokers in Bangladesh: Findings from the International Tobacco Control (ITC) Bangladesh wave 2 survey. Glob. Health Res. Policy.

[45] Macleod J., Hickman M., Bowen E., Alati R., Tilling K., Smith G. (2008). Parental drug use, early adversities, later childhood problems and children’s use of tobacco and alcohol at age 10: Birth cohort study. Addiction.

[46] Siahpush M., Borland R., Scollo M. (2003). Smoking and financial stress. Tob. Control.

[47] John R., Cheney M.K., Azad M.R. (2009). Point-of-sale marketing of tobacco products: Taking advantage of the socially disadvantaged?. J. Health Care Poor Underserved.

[48] Apollonio D., Malone R.E. (2005). Marketing to the marginalised: Tobacco industry targeting of the homeless and mentally ill. Tob. Control.

[49] Siahpush M., Yong H., Borland R., Reid J.L., Hammond D. (2009). Smokers with financial stress are more likely to want to quit but less likely to try or succeed: Findings from the International Tobacco Control (ITC) four country survey. Addiction.

[50] Sharma A., Lewis S., Szatkowski L. (2010). Insights into social disparities in smoking prevalence using Mosaic, a novel measure of socioeconomic status: An analysis using a large primary care dataset. BMC Public Health.

[51] Gilpin E.A., White M.M., Farkas A.J., Pierce J.P. (1999). Home smoking restrictions: Which smokers have them and how they are associated with smoking behavior. Nicotine Tob. Res..

[52] Kaleta D., Polanska K., Usidame B. (2015). Smoke-Free Workplaces Are Associated with Protection from Second-Hand Smoke at Homes in Nigeria: Evidence for Population-Level Decisions. Biomed. Res. Int..

[53] Kaleta D., Fronczak A., Usidame B., Dziankowska-Zaborszczyk E., Makowiec-Dąbrowska T., Wojtysiak P. (2016). Implementation of smoke-free homes in Poland. Int. J. Occup. Med. Environ. Health.

[54] Myung S.K., Seo H.G., Cheong Y.S., Park S., Lee W.B., Fong G.T. (2012). Association of sociodemographic factors, smoking-related beliefs, and smoking restrictions with intention to quit smoking in Korean adults: Findings from the ITC Korea Survey. J. Epidemiol..

[55] Willemsen M.C., de Vries H., van Breukelen G., Oldenburg B. (1996). Determinants of intention to quit smoking among Dutch employees: The influence of the social environment. Prev. Med..

[56] Wiltshire S., Bancroft A., Parry O., Amos A. (2003). I came back here and started smoking again: Perceptions and experiences of quitting among disadvantaged smokers. Health Educ. Res..

[57] Christiansen B., Reeder K., Hill M., Baker T.B., Fiore M.C. (2012). Barriers to effective tobacco-dependence treatment for the very poor. J. Stud. Alcohol Drugs.

[58] Fiore M.C., Jaen C.R., Baker T.B., Bailey W.C., Benowitz N.L., Curry S.J., Dorfman S.F., Froelicher E.S., Goldstein M.G., Healton C.G. (2008). Treating Tobacco Use and Dependence: 2008 Update.

[59] Kaleta D., Korytkowski P., Makowiec-Dąbrowska T., Usidame B., Bąk-Romaniszyn L., Fronczak A. (2012). Predictors of long-term smoking cessation: Results from the global adult tobacco survey in Poland (2009–2010). BMC Public Health.

[60] Aveyard P., Raw M. (2012). Improving smoking cessation approaches at the individual level. Tob. Control.

[61] Christiansen B.A., Reeder K.M., TerBeek E.G., Fiore M.C., Baker T.B. (2015). Motivating Low Socioeconomic Status Smokers to Accept Evidence-Based Smoking Cessation Treatment: A Brief Intervention for the Community Agency Setting. Nicotine Tob. Res..

[62] Franco L., Welsby D., Eccleston P., Furber S. (2011). A qualitative study about smoking cessation with clients of community service organisations that work with disadvantaged families. Health Promot. J. Aust..

[63] Christiansen B., Brooks M., Keller P.A., Theobald W.E., Fiore M.C. (2010). Closing tobacco-related disparities: Using community organisations to increase consumer demand. Am. J. Prev. Med..

[64] O’Brien J., Salmon A., Geikie A., Jardine A., Oakes W. (2010). Integrating smoking care in community welfare agencies to reach disadvantaged people: Findings from the Smoking Matters Project. Health Promot. J. Aust..

[65] McNeill A., Amos A., McEwen A., Ferguson J., Crogham E. (2012). Developing the evidence base for addressing inequalities and smoking in the United Kingdom. Addiction.

[66] Patrick D.L., Cheadle A., Thompson D.C., Diehr P., Koepsell T., Kinne S. (1994). The validity of self-reported smoking: A review and meta-analysis. Am. J. Public Health.

[67] Lawrence D., Mitrou F., Zubrick S.R. (2009). Smoking and mental illness: Results from population surveys in Australia and the United States. BMC Public Health.

[68] Siru R., Hulse G.K., Tait R.J. (2009). Assessing motivation to quit smoking in people with mental illness: A review. Addiction.

[69] World Health Organization (2014). Global Status Report on Noncommunicable Diseases 2014. Attaining the Nine Global Noncommunicable Diseases Targets.

[70] World Health Organization (2011). WHO Report on the Global Tobacco Epidemic, 2011—Warning about the Dangers of Tobacco. http://www.who.int/tobacco/global_report/2011/en/.

[71] International Agency for Research on Cancer (IARC) (2009). IARC Handbooks of Cancer Prevention, Tobacco Control, Volume 13: Evaluating the Effectiveness of Smoke-Free Policies.

[72] World Health Organization (2009). WHO Report on the Global Tobacco Epidemic, 2009—Implementing Smoke-Free Environments. http://www.who.int/tobacco/mpower/2009/en/.

