# Supplementary material for: Smoking Patterns and Smoking Cessation Willingness—A Study among Beneficiaries of Government Welfare Assistance in Poland
Source: Int J Environ Res Public Health. 2017 Jan 27;14(2):131. doi: 10.3390/ijerph14020131 (PMC5334685; doi:10.3390/ijerph14020131)
Supplement: Supplementary file 1 [file ijerph-14-00131-s001.pdf]

Supplementary Materials: Smoking Patterns and Smoking Cessations Willingness—A Study among Beneficiaries of Government Welfare Assistance in Poland

Katarzyna Milcarz, Teresa Makowiec-Dąbrowska, Leokadia Bak-Romaniszyn and Dorota Kaleta

Table S1. Distribution of adults ≥15 years old by selected demographic characteristics—GATS Poland, 2009–2010 *.

Variable	Number of Adults *n* (Overall *n* = 7840)	Percentage	(95% CI)
Gender			
Male	3867	47.7	(46.2–49.3)
Female	3973	52.3	(50.7–53.8)
Age (years)			
15–19	362	7.7	(6.8–8.6)
20–29	1306	19.5	(18.2–20.8)
30–39	1516	17.0	(16.1–18.0)
40–49	1348	16.6	(15.7–17.6)
50–59	1441	17.1	(16.0–18.2)
60+	1867	22.2	(21.0–23.5
Residence			
Rural	4012	38.0	(37.2–38.8)
Urban	3828	62.0	(61.2–62.8)
Up to 50,000	1441	40.1	(33.7–46.9)
50000–200,000	981	24.8	(19.6–30.9)
Over 200,000	1406	35.1	(29.1–41.5)
Education Level			
Primary	1630	20.0	(18.8–21.3)
Vocational	2092	24.8	(23.6–26.0)
Secondary	2951	40.0	(38.5–41.5)
High	1142	15.2	(14.1–16.3)
Employment status			
Hired employee employed in a company enterprise–based on employment contract or contract self–employed; owner or co–owner of a company or helping spouse	3530	45.2	(44.1–46.3)
Person keeping his/her own farm owner or co–owner of a farm or helping spouse on a farm	390	5.0	(4.5–5.5)
Pupil student	565	7.2	(6.6–7.8)
Person occupied with household keeping raising children/homemaker	526	6.7	(6.2–7.3)
Retiree	1878	24.1	(23.2–25.1)
Pensioner	480	6.2	(5.7–6.7)
Unemployed currently with no permanent job	433	5.6	(5.1–6.1)
Monthly income			
Less than 1000 PLN	2013	25.7	(24.7–26.7)
From 1000 –1500 PLN	1524	19.4	(18.5–20.3)
From 1501–2000 PLN	1050	13.4	(12.7–14.2)

From 2001–3000 PLN	809	10.3	(9.6–11.0)
Over 3000 PLN	547	7.0	(6.4–7.6)
I do not know	183	2.3	(2.0–2.6)
Refused	1714	21.9	(21.0–22.8)
Awareness of smoking health consequences			
Yes	7127	95.3	(94.8–95.8)
No	348	4.7	(4.2–5.2)
Exposure to environmental tobacco smoke			
At home	4879	62.4	(61.3–63.5)
Among respondents who work indoors and are exposed to tobacco smoke at work	1742	53.7	(52.6–54.8)

* Available from http://www.who.int/tobacco/surveillance/en_tfi_gats_poland_report_2010.pdf.
